# Five EMT‐related genes signature predicts overall survival and immune environment in microsatellite instability‐high gastric cancer

**DOI:** 10.1002/cam4.4975

**Published:** 2022-07-04

**Authors:** Mili Zhang, Can Cao, Xu Li, Qisheng Gu, Yixin Xu, Ziyan Zhu, Duogang Xu, Shanshan Wei, Haonan Chen, Yuqin Yang, Hugh Gao, Liang Yu, Jikun Li

**Affiliations:** ^1^ Department of General Surgery, Shanghai General Hospital Shanghai Jiaotong University School of Medicine Shanghai China; ^2^ Institute Pasteur of Shanghai, Chinese Academy of Sciences Shanghai China; ^3^ Department of General Surgery Shanghai General Hospital of Nanjing Medical University Shanghai China; ^4^ Department of Laboratory Animal Centre, Shanghai General Hospital Shanghai Jiao Tong University School of Medicine Shanghai China; ^5^ Department of Molecular and Translational Science Monash University Clayton Victoria Australia; ^6^ Department of Upper Gastrointestinal and Hepatobiliary Surgery, Monash Health Clayton Australia

**Keywords:** epithelial‐mesenchymal transition, gastric cancer, microsatellite instability‐high, prognosis, tumor microenvironment

## Abstract

**Background:**

Microsatellite instability‐high (MSI‐H) subgroup of gastric cancer (GC) is characterized by a high tumor mutational burden, increased lymphocytic infiltration, and enhanced inflammatory cytokines. GC patients with MSI‐H status have a good response to immune checkpoint blockade management. However, heterogeneity within the subtype and the underlying mechanisms of shaping tumor microenvironments remain poorly understood.

**Methods:**

RNA expression levels and clinical parameters of GC were obtained from The Cancer Genome Atlas (TCGA) and Gene Expression Omnibus (GEO) databases. The data were analyzed using single‐sample Gene Set Enrichment Analysis (ssGSEA), univariate Cox regression, multivariate Cox regression, and Least Absolute Shrinkage Selection Operator (LASSO) regression. In addition, multiplex immunohistochemistry (mIHC) was used in our clinical cohort for the tumor microenvironment study.

**Results:**

By ssGSEA and survival analysis, the EMT signaling pathway was identified as a representative pathway, which can stratify the patients with MSI‐H GC with significant survival predictive power. Then, a novel representative EMT‐related five‐gene signature (namely CALU, PCOLCE2, PLOD2, SGCD, and THBS2) was established from EMT signaling gene set, which sensitivity and specificity were further validated in the independent GEO database (GSE62254) cohort for disease outcome prediction. Based on public single‐cell data and in situ immunohistochemistry, we found that most of these five genes were abundantly expressed in cancer‐associated fibroblasts. Furthermore, patients with high or low risk divided by this five‐gene signature exhibited a strong correlation of the distinct patterns of tumor immune microenvironment. By mIHC staining of sections from 30 patients with MSI‐H status, we showed that the patients with better prognoses had the increased infiltration of CD8^+^ cells in the primary tumoral tissue.

**Conclusion:**

Our study developed a simple five‐gene signature for stratifying MSI‐H GC patients with survival predictive power.

## Graphical abstract

Zhang et al. developed a simple five‐gene signature for stratifying MSI‐H GC patients. The gene signature based on EMT‐related genes is helpful in predicting the prognosis of MSI‐H GC patients and, to some extent, reflects the immune microenvironment of patients.

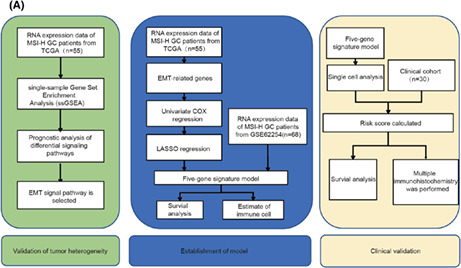

## 1 INTRODUCTION

Gastric cancer (GC) is one of the most common causes of cancer death around the world.^1^ Patients of MSI‐high subtype account for a small fraction (only 8%–20%), which may vary across different cancers.^2^, ^3^, ^4^ This subgroup of GC is characterized by a high tumor mutational burden (TMB) with the expression of upregulated immunogenic neoantigen peptides, which is associated with increased lymphocytic infiltration and enhanced inflammatory cytokines.^5^ Emerging evidence demonstrated that patients with MSI‐H subtype of tumor are more likely to benefit from the management of immune checkpoint blockade (ICB) therapy.^6^, ^7^

However, the molecular and cellular heterogeneity within the tumor microenvironment of MSI‐H tumors remains poorly characterized. We performed immune‐genomic molecular analyses to characterize a guiding biomarker for the stratification of MSI‐H GC, facilitate the selection of the patients who respond to these treatments, and provide support to move the field toward customized immunotherapeutic strategies in MSI‐H GC. A few efforts have been devoted to the molecular subtyping of MSI‐H tumors based on gene expression profiling.^8^, ^9^ For example, Yang et al. used non‐negative matrix factorization (non‐NMF) based consensus clustering to define GC patients with MSI‐H status into two groups with different prognoses. They find that immunosuppressive factors are enriched in one subgroup compared with another, which may be related to the poor prognosis of these patients.^9^ However, this study relied on retrospective analysis from public databases and lacked the evidence of inherent observations of the tumor microenvironment.

Epithelial‐mesenchymal transition (EMT) plays a vital role in tumor growth and metastasis, a reversible dynamic process.^10^ EMT has critical implications for clinical oncology, as it could be a novel predictive biomarker of clinical response for ICB.^11^, ^12^ Since transcriptional data can be easily obtained from online data centers, establishing genetic characteristics of cancer mechanisms is an active research field.^13^, ^14^, ^15^ Considering EMT status has been proved to be a prognostic marker of GC, EMT‐related genes are a significant source for predicting the prognosis of MSI‐H GC patients.^16^

In this study, we conducted a single‐sample Gene Set Enrichment Analysis (ssGSEA) using transcriptomic data from independent cohorts to stratify MSI‐H GC subtypes with different risk scores. Intriguingly, we identified five EMT‐related genes signature associated with the prognosis of MSI‐H GC and enriched stroma recruitment. Coupling multiplex immunohistochemistry (mIHC) staining and computational analysis of independent cohorts, the upregulation of these five EMT‐related genes signature indicated a suppressive tumor immune microenvironment characterized by the reduction of CD8^+^ T cell infiltration. Our study provided a novel insight to understand the heterogeneity of MSI‐H GC better. The five‐gene signature may improve the ability to predict prognosis in GC patients and respond to ICB.

## 2 MATERIALS AND METHODS

### 2.1 Data collection

RNA‐Seq data (FPKM) and clinical information data of Stomach Adenocarcinoma (STAD) were retrieved from TCGA database (https://portal.gdc.cancer.gov/), and microarray data (GSE62254) was acquired from Gene Expression Omnibus (GEO) repository (https://www.ncbi.nlm.nih.gov/geo/). Patients without survival information were removed from further evaluation. All gene expression data were uniformly normalized. Data were analyzed with R (v 4.0.0) and R Bioconductor packages.

### 2.2 Human GC biopsy

In this study, biopsies of 30 GC tissues from patients with negative MLH1 staining were collected from Shanghai General Hospital. All biopsies were continuous samples obtained from surgical resection and immediately processed into formalin‐fixed and paraffin‐embedded tissues to produce tissue arrays. In addition, all samples were confirmed as GC by two pathologists, and basic information including clinicopathological features of patients was collected. This study was approved by the ethics committee of the Shanghai General Hospital. Informed consent was obtained from every GC patient participating in this study.

### 2.3 EMT‐related genes list

This list contained 200 EMT‐related genes, which were accessed from the HALLMARK_EPITHELIAL_MESENCHYMAL_TRANSITION gene set of the Molecular Signatures Database (MSigDB).^17^ The complete gene list was contained in Table S1.

### 2.4 Principal component analysis

Principal Component Analysis (PCA) is an algorithm technique that helps picture the information as an eminent multivariate statistical method. PCA can indicate the similarity or difference among samples and reveal which variables will affect the similarity or difference. In addition, PCA can detect the sample pattern of grouping. We used hallmark classes (oncogenic, immune, stromal, stress, and other) from Jiménez‐Sánchez to evaluate the significance of differences between the means of the loadings defined on the PCA dimensions of interest.^18^

### 2.5 single‐sample gene set enrichment analysis

single‐sample Gene Set Enrichment Analysis algorithm belongs to a special GSEA. It mainly proposes an implementation method for a single sample unable to conduct GSEA. It also calculates the rank value of each gene according to the expression profile file and then carries out subsequent statistical analysis. The final result is that each example has its score under the corresponding background gene set. The modification of standard GSEA, ssGSEA, was performed on RNA measurements for each sample using the GSVA package (v 1.36.2) with R.

#### 2.5.1 Least absolute shrinkage and selection operator regression

Least absolute shrinkage and selection operator (LASSO) regression analysis was conducted by the glmnet R package (v 4.1–3). We performed it to determine the potential predictors with non‐zero final elimination coefficient, so as to avoid overfitting the model and select the optimal genes.

### 2.6 Survival analysis

The survival difference between the subgroups was tested by the Kaplan–Meier (KM) and log‐rank methods with the functions Survfit and Survdiff in the survival package for R (v 3.1.12). A Cox univariate model was applied to compare the subtypes with the function Coxph in the R package survival. In addition, the receiver operating characteristic (ROC) curve is conducted by the ROCR R package (v 1.0–11) to calculate the area under the curve (AUC) value of the ROC curve of each prediction model to further evaluate the efficiency and accuracy of the model. *p* value <0.05 was considered significant.

### 2.7 Immune microenvironment assessment

To quantify the proportion of immune cells in samples, we used CIBERSORTx algorithm^19^ and LM22 gene signature, including T cells, B cells, macrophages, natural killer cells subsets, and so on. We prepared gene expression profiles as standard files and then upload them to CIBERSORTx (http://cibersortx.stanford.edu/).

### 2.8 Multiplex immunohistochemistry staining

Four‐micron slices were cut from the Paraffin block of patient tissue onto charged slides, and slides were baked at 60°C for 1 h. Deparaffinization twice with xylene for 10 min and then stained by 100%, 90%, and 70% ethanol for 10 min each. Slides were washed in deionized water for 2 min, followed by neutral buffered formalin for 30 min. The Opal manual kit (PerkinElmer) was used according to the manufacturer's instructions. After each antigen retrieval, slides were stained with antigen‐specific primary antibodies [PD‐L1(13684s), CD8α(70,306s), and FOXP3(98,377s), Cell Signaling Technology, Danvers, MA, USA, CD163(16646), CD80/b7‐1(66406), Pan‐keratin(24611), CALU(17804‐1‐AP), PLOD2(21214‐1‐AP), Proteintech, Rosemount, IL, USA, THBS2(40425), Signalway Antibody, College Park, MD 20740, USA] followed by Opal Polymer (secondary antibody). Application of the Opal TSA created a covalent bond between the fluorophore and the tissue at the site of the horseradish peroxidase (HRP). Each antigen retrieval step was performed using AR9 antigen retrieval buffer, which allowed for the removal of prior primary and secondary antibodies while the fluorophore remained covalently bonded to the tissue antigen. This allowed for the use of the same host species antibody while also amplifying the signal.

### 2.9 Multiple immunohistochemical staining analysis

All slides were scanned and imaged by Vectra Polaris (Perkin Elmer) and Nikon C1 confocal system (Tokyo, Japan). All images were analyzed by ImageJ software (NIH, Bethesda, MD, USA). Since there was much deviation, we presented the results as log10 expression/mm^2^ based on the value we got from ImageJ.

### 2.10 Statistical analysis

Continuous variables and categorical variables (percentage and frequency) were analyzed by independent *t*‐test, chi‐square test, or two‐tailed Fisher exact test, respectively. All statistical analyses were performed in R software (version 4.0.0) software. All statistical tests with *p* < 0.05 were statistically significant.

## 3 RESULTS

### 3.1 Analysis process overview

**FIGURE 1:**
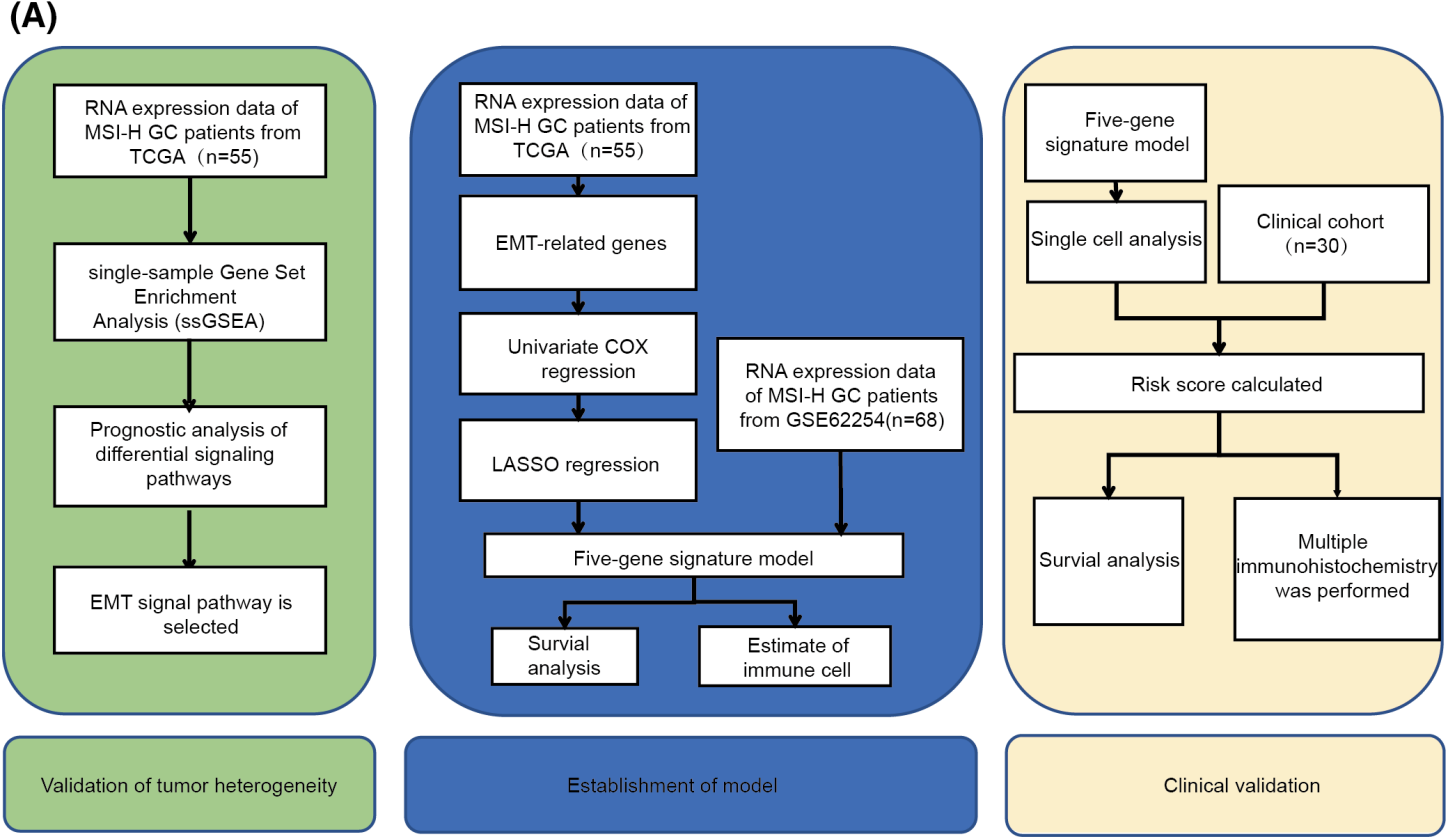
The flow chart of prognosis model of MSI‐H GC patients. (A) Main process of model construction and relevant steps of verification of the five‐gene signature in MSI‐H GC patients. The whole process is divided into three parts. The first part is the verification of tumor heterogeneity (green), the second part is the establishment of the model (blue), and the third part is the verification of the clinical cohort (orange).

The flow chart of the study design is shown in Figure 1. Gene expression by RNA sequencing of 55 GC patients with MSI‐H status in the TCGA dataset was collected and subjected to ssGSEA using MSigDB Hallmark gene sets for heterogeneity analysis, which can be seen in the green part. In the blue part of the flow chart, we identified five EMT‐related genes as biomarkers most predictive of OS using Lasso regression analysis, and the five‐gene signature was validated in an independent RNAseq cohort (GSE62254). Finally, we constructed a stable prognostic model through bioinformatics and identified by multiplex immunohistochemistry (mIHC) staining in the clinical cohort (*n* = 30) in the orange part.

### 3.2 Transcriptome heterogeneity in MSI‐H GC patients

**FIGURE 2:**
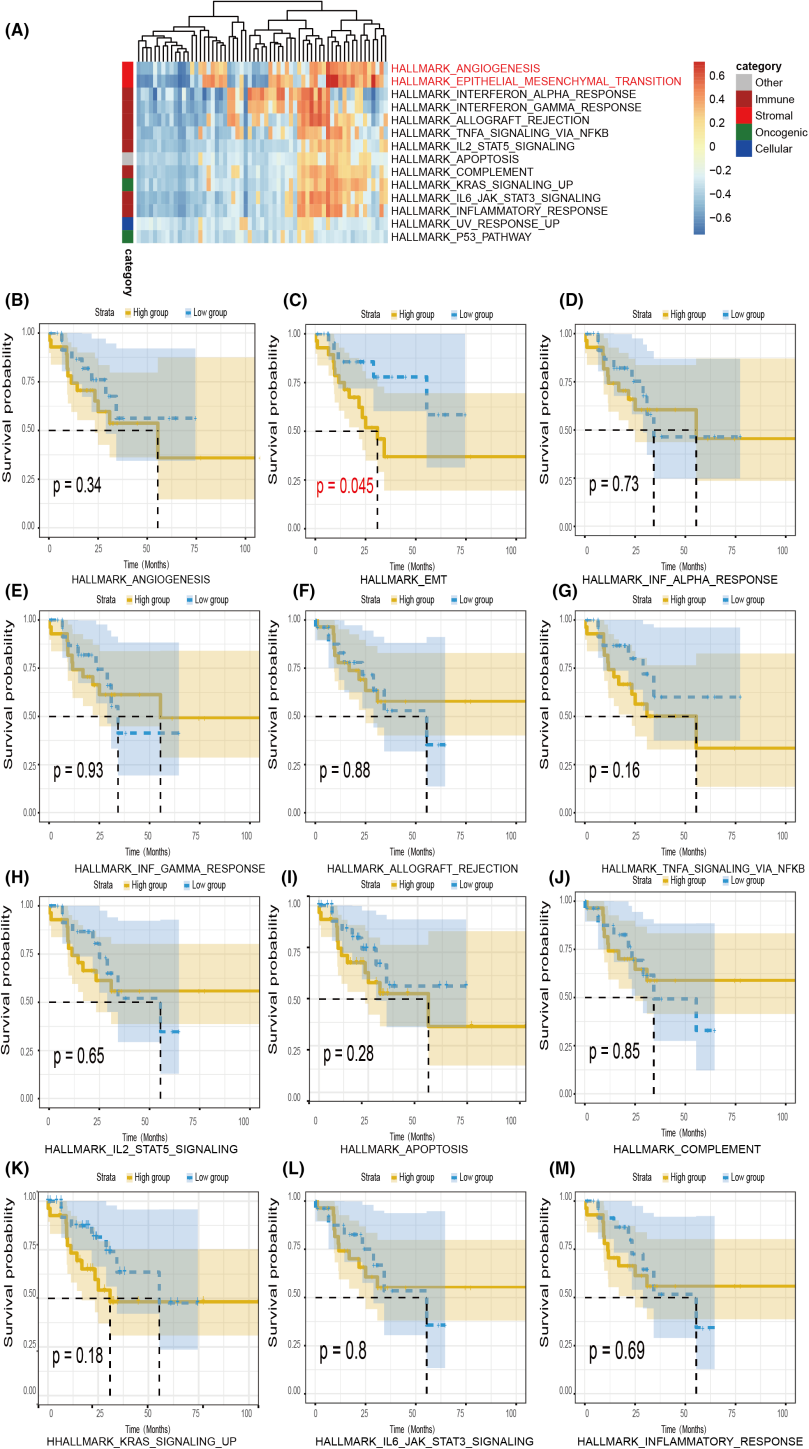
Identification of transcriptome heterogeneity. (A) Heatmap displaying the scores and relationship between cancer hallmark gene sets. (B–M) Kaplan–Meier curves were used for survival analyses between patients who were stratified into the high or low group.

In order to study the heterogeneity of gastric cancer patients with MSI‐H, we analyzed the transcriptome of TCGA MSI‐H GC patients provided. Compared with the previous transcriptome analysis, the study of the marker gene set can more clearly reveal that tumors from different patients have similar signaling pathways activation modes.^9^ To focus on well‐defined biological processes and signal transduction pathways, we used MSigDB hallmark gene sets for ssGSEA (Figure S1A). Jimenez‐Sanchez et al. divided 50 signaling pathways into five categories: Oncogenic, cellular, immunity, stromal, and others.^18^ Concurrently, the heatmap of the gene sets shows showed that Angiogenesis and EMT pathways reflected the most heterogeneity among MSI‐H patients (Figure 2A). Differential enrichment of immune‐related gene sets, such as Interferon alpha and gamma, TNF, and JAK_STAT3 pathways, also exhibited many variations between the samples (Figure 2A). To further analyze the inherent relationship between OS and different pathways, we conducted survival analysis by dividing the patients into two groups according to the results of ssGSEA based on the median score (Figure 2B–M). EMT pathway was the only well‐defined gene set that had a significantly negative association with OS of MSI‐H GC patients in TCGA (Figure 2C). Taken together, this data suggested that EMT‐related gene enrichment variation represented the heterogeneity that exists in MSI‐H tumors, which may convey a predictive power for patient OS.

### 3.3 Identification of survival‐related genes in MSI‐H GC

**FIGURE 3:**
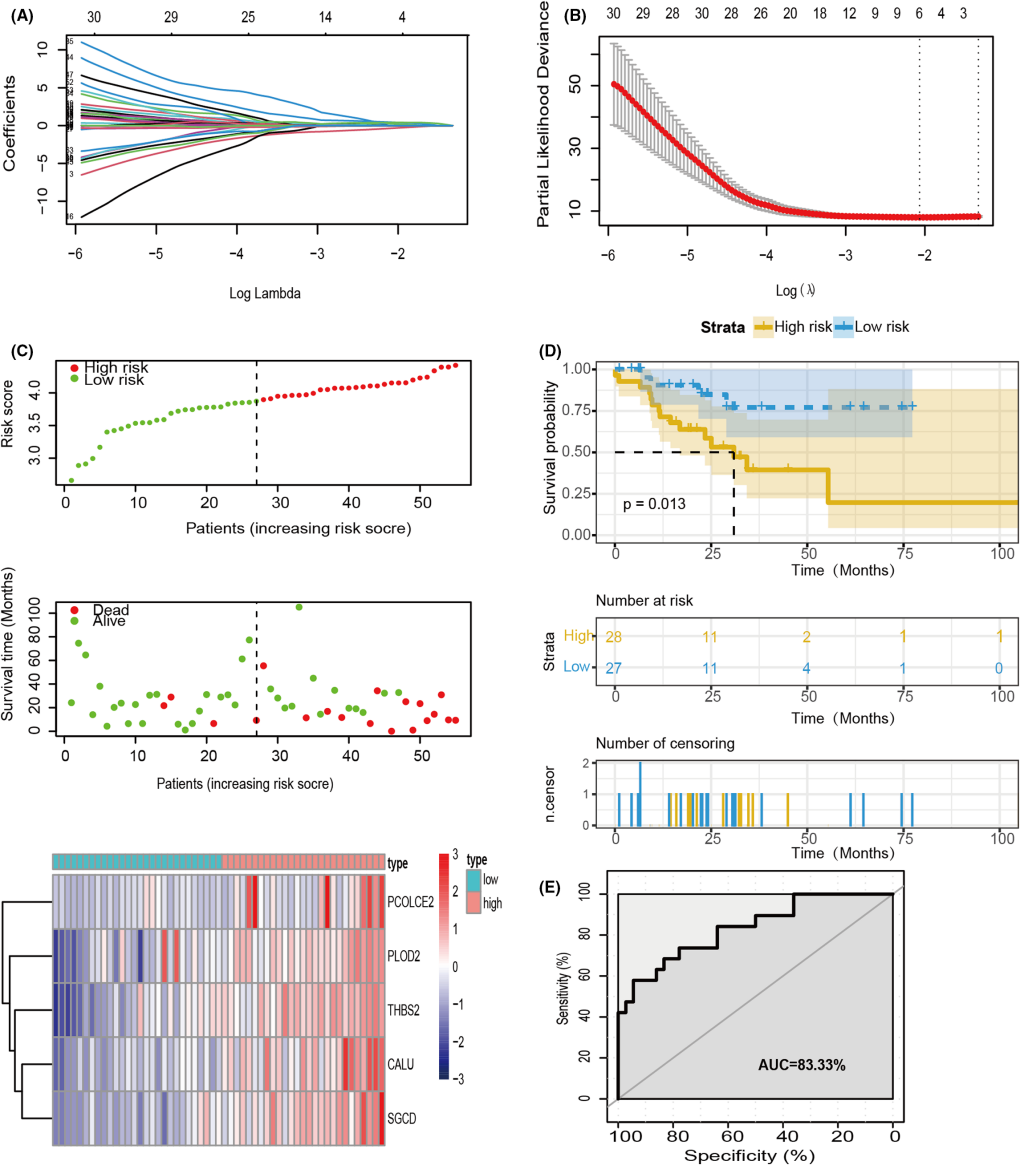
Prognostic value of TCGA MSI‐H GC training cohort prediction model. (A) A LASSO coefficient profile plot was drawn against the optimal lambda. (B)Selection of the perfect parameter (lambda) in the model; minimum criteria were drawn at the optimal values. (C) The distribution of the survival status and risk score of MSI‐H GC patients. Heatmap of the five genes expression profiles between the low‐ and high‐risk groups. (D) The top part shows the Kaplan–Meier curves; the central section shows the variations of living patients; the underneath shows the variations of censoring with time. (E) ROC curves analysis of the five genes signature.

To explore the possible relationship between EMT and prognosis in patients with MSI‐H, we adopted univariate Cox analysis to screen survival‐related genes in the TCGA cohort. From 200 genes from the EMT gene set,^17^ only 63 genes had a significant predictive value for OS when assessed alone (*p* < 0.05, Figure S1B). Then, we performed LASSO regression to choose the optimal OS‐related genes with nonzero coefficients (Figure 3A,B). Multivariate Cox regression analysis was also performed to identify the correlation between these genes and patient survival and obtain corresponding coefficients (Table S2). As a result, EMT‐related five‐gene signature containing CALU, PCOLCE2, PLOD2, SGCD, and THBS2 were acquired as a superior biomarker for predicting survival, suggesting that the combination of these five genes, rather than each gene in isolation, showed the optimal value and can be utilized to build a prognostic model for patient stratification. The risk score of each patient was calculated according to the following formula: Risk score = expression level of CALU* − 0.08624808 + expression level of PLOD2* 0.48197972 + expression level of PCOLCE2 × 0.55736195 + expression level of SGCD* 0.68391051 + expression level of THBS2 × 0.24390893 (Table S2). According to the median risk score, MSI‐H GC patients were divided into high‐ and low‐risk groups. The survival status and the distribution of risk scores of MSI‐H GC patients were shown in Figure 3C. In addition, the heatmap showed different expression levels of these five genes in these two groups (Figure 3C). The KM curve demonstrated that MSI‐H GC patients with low‐risk scores had a longer survival time than those with high‐risk scores (*p* < 0.05, Figure 3D). The areas under the curve (AUCs) for the risk score in predicting OS were 0.8333 (Figure 3E). Similarly, we respectively evaluate the outcomes and AUCs in five genes (Figure S2A–J). To sum up, the above results showed that the risk score from the TCGA dataset has the immense ability to predict the OS of MSI‐H GC patients.

As MSI‐H status is often associated with hypermethylation induced MLH1 inactivation and BRAF mutation,^20^ we also studied whether these molecular events contributed to a poorer prognosis in GC patients with MSI‐H. It appeared to have no significant correlation between loss of MLH1 expression or BRAF mutation with a high‐risk score of this signature (Figure S3A). Altogether the above results showed that the risk score from the TCGA dataset has the immense ability to predict the OS of MSI‐H GC patients.

### 3.4 External validation of the five‐gene prognostic signature

**FIGURE 4:**
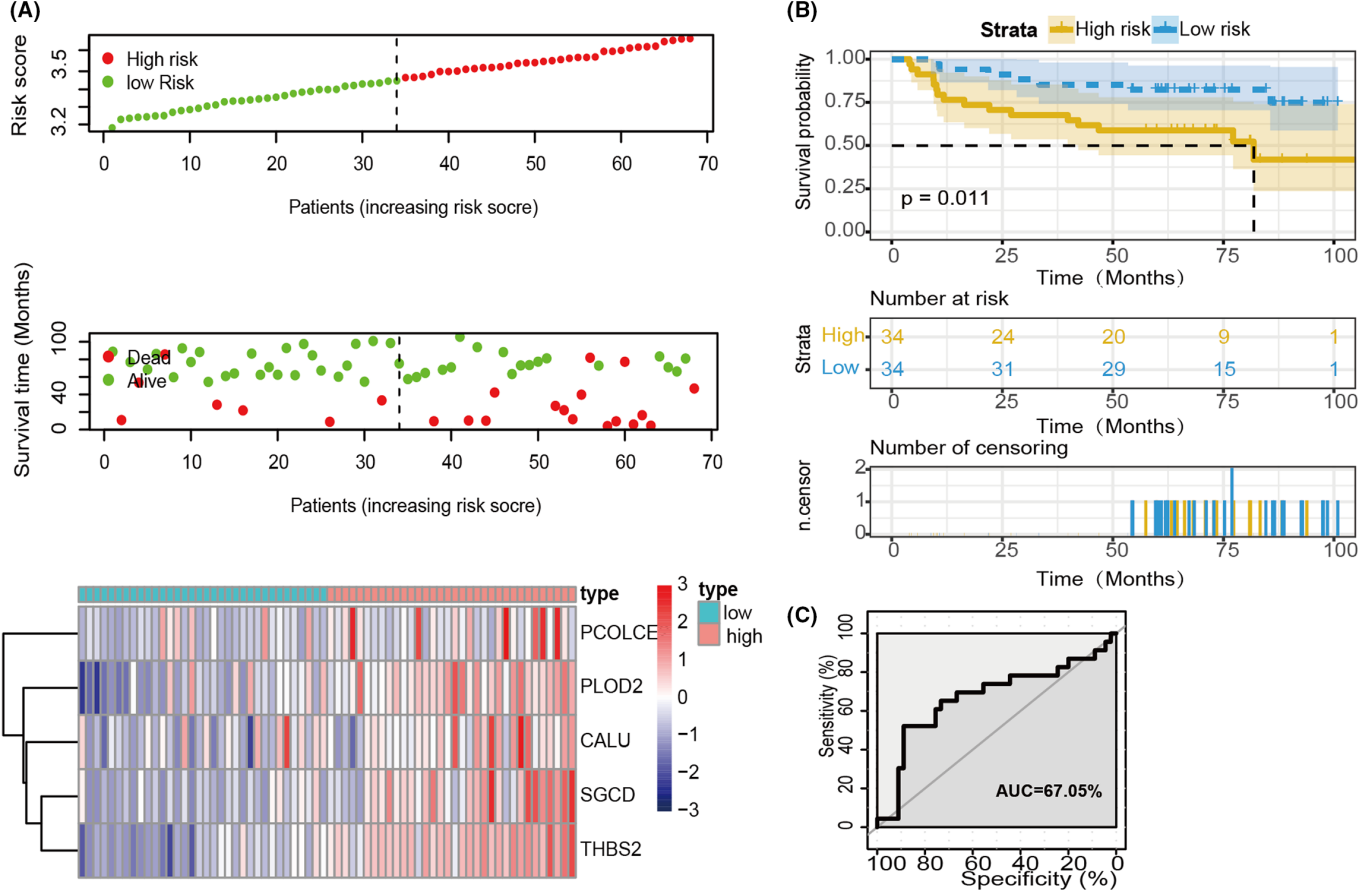
Prognostic value of GEO validation cohort prediction model. (A–C) GSE62254 Validation cohort displays patients with high‐risk scores have worse outcomes than those with low‐risk scores, which was the same as the results of the training cohort.

Next, we validated the five‐gene signature in the training cohort of the GEO database to confirm our findings by using the same model in the testing group. The patients from GSE62254 (*n* = 68), which were highlighted with MSI‐H, were divided into low‐ and high‐risk subtypes by calculating their median risk score (Figure 4A). In agreement with the subgroup from TCGA, the KM survival curves revealed that the high‐risk group had a shorter survival rate than the low‐risk group (Figure 4B), consistent with the training group. In addition, we also assessed the predictive ability of this signature to significantly stratify MSI‐H patients, as determined by the ROC curve of the model with the AUC value of 67% (Figure 4C).

### 3.5 Quantification of different markers has significance in distinguishing types of MSI‐H GC patients

**FIGURE 5:**
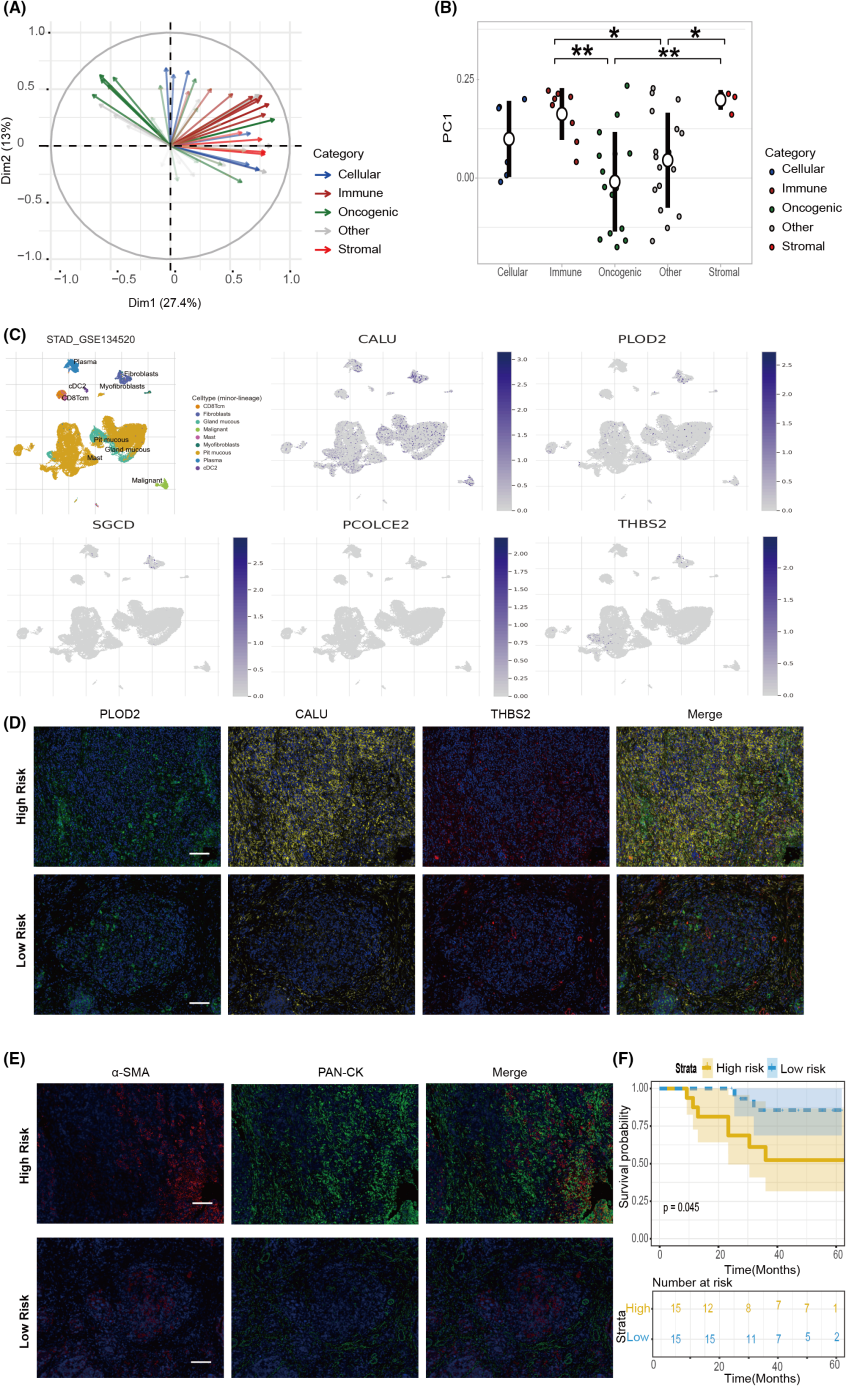
Validation of five‐gene signature in MSI‐H patients. (A) Principal component analysis (PCA) calculated using single sample genome enrichment analysis (ssGSEA) calculated based on TCGA RNA‐seq. (B) Principal‐component feature loadings (direction and magnitude) from A shown. (C) The analysis results of five genes in TISCH (http://tisch.comp‐genomics.org/). (D) Immunofluorescent staining for PLOD2 (green)/DAPI (blue), CALU (yellow)/DAPI (blue) and THBS2 (red)/DAPI (blue) of sections from MSI‐H GC patients' sections. (E) Immunofluorescent staining for PAN‐cytokeratin(red)/DAPI(blue), α‐SMA (green)/DAPI(blue) of the same place in the series of sections. Scale bar, 100 μm. (F) Evaluation of the prognostic value of the predictive model in the MSI‐H GC patients. *p < 0.05, **p < 0.01, and ***p < 0.001.

To further explore the spatial expression pattern of the five genes in the tumor microenvironment, we performed immunofluorescence staining in our samples identified as MSI‐H status collected from the surgery. Firstly, PCA showed that the majority of gene sets were enriched (27.4%); From the first two significant components can be explained by oncogenic, immunity, and stromal gene set (Figure 5A,B). This may suggest that the five genes we selected are largely expressed in specific cells, not all tumor cells. We analyzed the five genes with the help of the TISCH (http://tisch.comp‐genomics.org/) single‐cell analysis database (GSE134520) to characterize specific cell populations with abundant expression of these genes.^21^ Except that PCOLCE2 did not express in any cells, CALU, PLOD2, SGCD, and THBS2 were expressed in fibroblasts of GC tissue. While CALU and PLOD2 expression were ubiquitously observed in various cell types, THBS2 has limited expression in mast cells and fibroblasts (Figure 5C). By coupling with the analysis of scRNA sequencing data of other types of human cancer,^22^ we confirmed that these genes were often abundantly expressed in fibroblasts (Figure S4A–E), especially in colorectal cancer. Consistently, while we observed the widely positive staining of CALU in tissue from patients, high expression of PLOD2 was shown in the tumoral with few THBS2 positive stained cells (Figure 5D). On the contrary, PLOD2 and THBS2 were significantly downregulated in tissue with low‐risk scores (Figure 5D). Therefore, we selected CALU, PLOD2, and THB2, which were enriched in cells, as our staining markers. We counted the numbers of these markers in different patients/mm^2^. According to the results, we divided the patients into high‐ and low‐risk groups according to the mentioned formula: Risk score = expression level of CALU* − 0.08624808 + expression level of PLOD2 × 0.48197972 + expression level of THBS2 × 0.24390893 (Table S3). Meanwhile, tumors with high‐risk scores determined by five‐gene signature demonstrated increased stromal compartment labeled with α‐SMA(α‐smooth muscle actin) antibody in the section as shown in (Figure 5E). At the same time, we compared the prognosis of the two groups, and the results showed that consistent with our previous model results, the low‐risk group had a better prognosis (Figure 5F).

### 3.6 Different immune‐phenotypes related immunotherapy response

**FIGURE 6:**
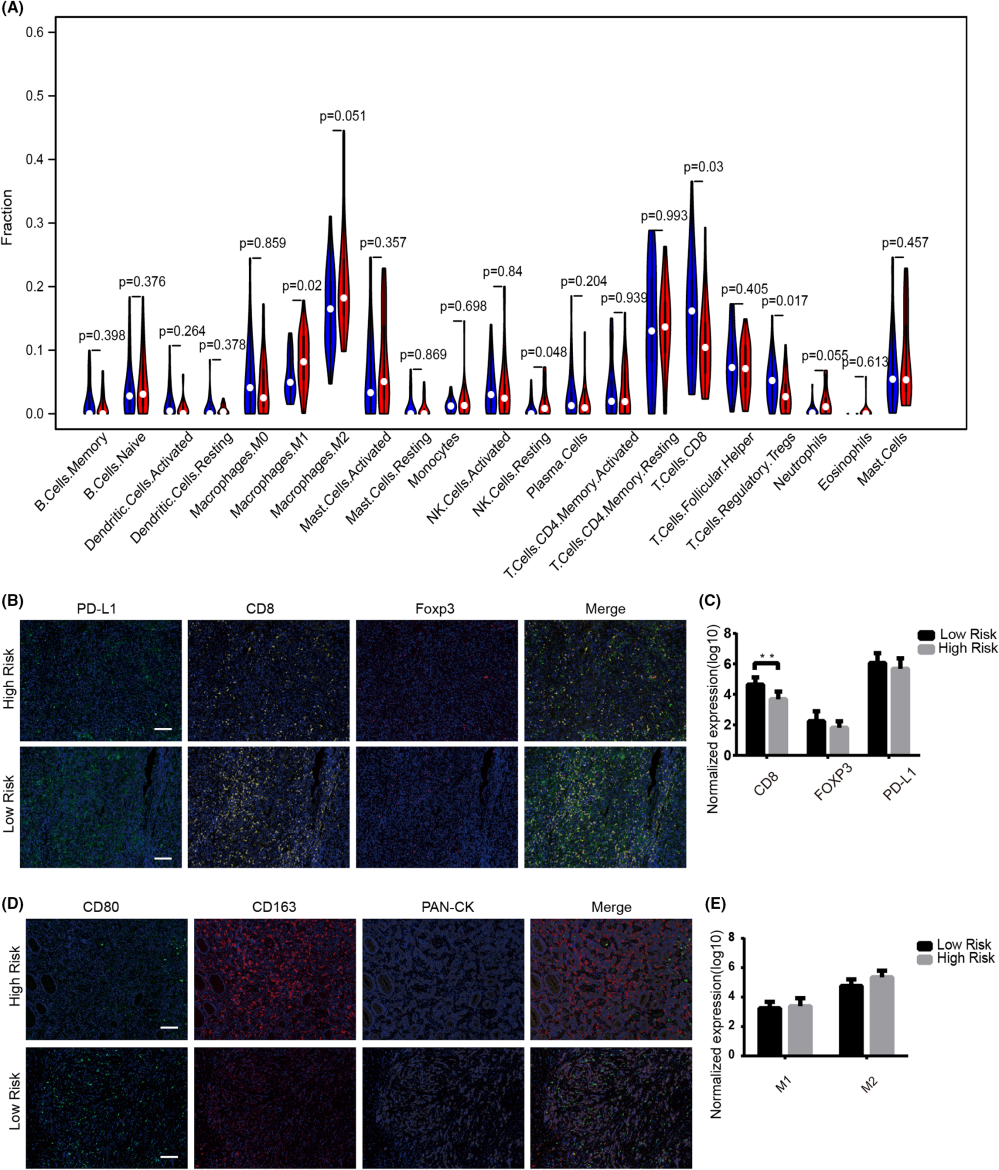
Analysis of the relationship between immune infiltration and five genes. (A) The distribution of various immune cell infiltration in TCGA high‐risk group and low‐risk group was analyzed by violin diagram. (B) Representative image of CD8^+^ T cells, FOXP3^+^ Tregs, and PD‐L1^+^ cells distribution in MSI‐H patients' samples from low‐ and high‐risk groups. Immunofluorescent staining for PD‐L1(green)/DAPI (blue), CD8(yellow)/DAPI (blue) and Foxp3(red)/DAPI (blue) of sections from MSI‐H patients. (C) Normalization expression(log10) of the number of CD8^+^ T cells, FOXP3^+^ Tregs, and PD‐L1^+^ cells per mm2. (D) Representative image of CD8^+^ M1 cells, CD163^+^ M2 cells, and PAN‐CK^+^ cells distribution in MSI‐H GC patients' samples from low‐ and high‐risk groups. Immunofluorescent staining for CD80 (green)/DAPI (blue), CD163 (red)/DAPI (blue) and PAN‐cytokeratin (gray)/DAPI (blue) of sections from MSI‐H GC patients. (E) Normalization expression(log10) of the number of M1 cells, M2 cells per mm^2^. Scale bar, 100 μm. *p < 0.05, **p < 0.01, and ***p < 0.001.

To estimate immune cell type abundances from bulk tissue transcriptomes, we used CIBERSORTx^19^ algorithm to characterize the distinct immune profiling of MSI‐H GC patients with low‐ and high‐risk scores. We compared the different levels of immune cell infiltration between low‐ and high‐risk groups. Violin plot revealed the levels of various immune cell infiltration. As shown in Figure 6A, the fraction of regulatory T cells (Tregs) and CD8 positive T cells were significantly higher in the low‐risk group than in the high‐risk group, whereas M1 cells in the low‐risk group were downregulated than that of the high‐risk group (Figure 6A). To further explore the evidence of our computational observation, mIHC staining was conducted in the FFPE section from our own collected biopsies of 30 GC patients with MSI‐H status. The spatial distribution of CD8^+^ T cells, PD‐L1^+^ cells, and FOXP3^+^ cells in the primary tumoral tissue were labeled by optimized and specific antibodies (Figure 6B). while there was no statistical significance in PD‐L1^+^ cells and FOXP3^+^ cells among different groups, CD8 positive cells exhibited increased positive cell number and expression intensity in the low‐risk group (*p* < 0.05, Figure 6C), which is consistent with the results of CIBERSORTx. In addition, we sought to visualize epithelial cells, M1 and M2 macrophages in the tumor microenvironment with PAN‐CK, CD80, and CD163 specific antibodies as the biomarkers. Despite no significant difference, we observed an increased cell number of M2 macrophages positively stained by CD163 in the high‐risk group (Figure 6D,E). These results indicated that the characterization of tumor heterogeneity by transcriptomic analysis in MSI‐H bulky tissue could be validated by internal observation of this disease's tumor immune microenvironment of an independent patient cohort.

## 4 DISCUSSION

MSI‐H status often indicates a better prognosis in colorectal cancer and GC.^23^, ^24^, ^25^ These tumors are characterized by accumulated non‐silent mutations, leading to increased exposure to neoepitopes and immune recognition.^26^ As a few clinical trials in MSI‐H GC and colorectal carcinoma patients using pembrolizumab showed promising results, treatment using PD‐1 blockade has been approved by FDA as first‐line management for the cancer patient with MSI‐H status regardless of cancer type.^27^, ^28^ However, some studies revealed that MSI‐H patients displaying the heterogeneous distribution of microsatellite status might contribute to conferring a poorer response to PD‐1 blockade.^9^, ^29^, ^30^, ^31^ Thus, an in‐depth molecular characterization of MSI‐H GC is required to identify transcriptomic traits of non‐responders with MSI‐H. By ssGSEA analysis, we first stratified MSI‐H GC patients with different risk scores with predictive power on patient outcome. Five EMT‐related genes signature was further identified as a proxy representative of the entire EMT gene set with the comparable capacity of predicting prognosis.

EMT has been identified as a critical driver of embryonic development, tissue fibrosis, wound healing, tumorigenesis, and metastasis.^12^, ^32^ As previously described by many studies, patients with enriched EMT‐related gene expression are associated with a poorer prognosis in many human cancers.^33^, ^34^, ^35^ In our study, the high‐risk group we identified also has the characteristics of EMT signal pathway activation and a worse prognosis, which is consistent with the previous study (Figures 3 and 4). A mesenchymal subtype of GC (defined by a positive EMT signature) often showed increasing resistance to radio‐ and chemotherapeutics and poor response to ICB treatment.^36^, ^37^, ^38^, ^39^ Intriguingly, PD‐1 blockade‐induced tumor immunity was inactivated in GC patients with the enriched EMT signature regardless of elevated level of the immune infiltrates, suggesting a complex influence of EMT‐related factors on tumor immune microenvironment.^11^, ^40^ We also found that pathways involved in inflammation, such as interferon response signaling pathways, were also enriched in patients with higher risk scores, albeit with no statistical significance in survival prediction (Figure S1A). This indicates that EMT often occurs concurrently with deregulated immune‐related signaling pathways in the tumor microenvironment.^41^ A positive feedback loop between different types of cells that interact with each other leads to the induction of immunosuppressive substances release of immune cells, which promotes tumor invasion and metastasis.^42^

Another finding in our study is the identification of a five‐gene signature for OS of MSI‐H GC patients. Single gene features or clinical factors are easily influenced by many factors, which makes them difficult to become reliable prognostic markers. We identified a combination of five genes (CALU, PCOLCE2, PLOD2, SGCD, and THBS2) with superior prognostic value for this subgroup than a single gene. CALU (Calumenin), as a pivotal gene of the EMT process, has been reported to directly link to the cancer metastasis of a variety of cancers.^43^, ^44^, ^45^ CALU may be involved in tumor microenvironment remodeling because of its close association with CD8 cells and macrophages.^46^, ^47^ PLOD2 (2‐oxoglutarate 5‐dioxygenase 2), an intracellular enzyme belonging to the PLOD family, plays a critical role in collagen modification, cell migration, and pulmonary metastasis with no impact on primary tumor growth.^48^, ^49^ Lewis et al. demonstrated expression of PLOD2, as a collagen matrix crosslinker, affected the tumor microenvironment of mechanical and chemical properties.^50^ Wang et al. introduced the role of PLOD2 in drug resistance in GC.^51^ Meanwhile, THBS2 belongs to the platelet reactive protein (THBS/TSP) family of five calcium binding stromal cell glycoproteins THBS1‐ THBS5, which mainly inhibits angiogenesis and negatively regulates MMP‐2 and MMP‐9. It has been shown that the downregulation of THBS2 is closely related to adverse survival in non‐small cell lung cancer, lung adenocarcinoma, and liver cancer.^52^, ^53^, ^54^ In the study of Ng et al., THBS2 deficient cancer stem cells give rise to hepatocarcinogenesis through histone H3 modification, promoting collagen degradation and decreasing matrix hardness and metastatic dissemination.^52^ In addition, PCOLCE2 (procollagen C‐endopeptidase enhancer 2), a collagen‐binding protein, was also considered as a mediator of the protein matrix.^55^, ^56^ However, few studies regarding the gene SGCD, a component of the sarcoglycan complex in cancer biology, have been previously reported.^57^ The increased expression of these five genes in our study appeared to link to the enrichment of cancer‐associated fibroblast based on the analysis of single‐cell sequencing data (Figure 5C). Functional studies of these genes in the context of cancer‐associated stromal cells warranted future investigations.

In human colorectal tumors, the expression of EMT markers is significantly correlated with adverse clinicopathological features and MSI, the EMT process of MSI tumors is not only related to TGF‐β but also depends on the recruitment of ERK.^58^ In mucosal melanoma cells, CALU deficiency has been shown to inhibit phosphorylation of ERK and is regulated by mir‐let‐7b and mir‐let‐7c.^59^ CALU may play a unique role in the EMT process of MSI tumor patients by affecting the function of ERK. Meanwhile, a study showed that PLOD2 is critical in TGF‐ β induced EMT progression, and the depletion of PLOD2 might inhibit TGF‐β1‐induced phenotypic EMT‐like changes by preventing β‐catenin from entering the nucleus.^60^ It suggests that the deletion of PLOD2 may hinder the EMT process in MSI‐H GC patients. In our model, high expression of PLOD2 patients tends to have higher risk scores, a worse prognosis, and the possibility of EMT signaling pathway activation. Moreover, in the study of Deng, PLOD3, as a member of the PLODs family, is considered to be related to immune cell infiltration and genomic instability in colon adenocarcinoma, which makes us wonder whether PLOD2 also plays the same role in MSI GC.^61^

In this study, we used the four‐color mIHC technique to visualize the immune environment of MSI‐H GC patients. The evidence of the in situ observation supported the notion that patients with increased EMT signatures were associated with a suppressive immune microenvironment (Figure 6A). Consistent with the previous findings from Dongre et al., there was still a trend that the high‐risk group has the upregulation of PD‐L1 expression (Figure 6B,C), despite no statistical difference.^62^ The aggregation of macrophages was also suggested as a key player in promoting the process of EMT (Figure 6D,E), as previously described in the interaction with stromal cells involved in prognosis‐related drug resistance, cancer progression, and failed immunosurveillance.^63^, ^64^ However, the role of tumor‐associated macrophages and their interaction with the CAFs with high expressed 5‐gene signature in the tumor microenvironment are worth further exploring.^65^

However, our study has several limitations. First, the group size of the patient cohort is small as the low incidence of MSI‐H GC. To overcome the shortage, further studies may be conducted by integrating all cancer types with a high frequency of MSI‐H, such as colorectal cancer and endometrial cancer. Second, a multicenter, prospective, and more extensive clinical cohort should be taken into consideration. Thirdly, the functions of these five genes in MSI‐H GC have not been fully investigated in an experimental setting.

## 5 CONCLUSION

Our study revealed that MSI‐H GC could be divided into two subtypes with distinct outcomes based on the enrichment of the EMT signaling pathway. The EMT pathway‐derived 5‐gene signature, namely CALU, PCOLCE2, PLOD2, SGCD, and THBS2, has a powerful prediction capacity for OS, facilitating patient stratification of GC patients with MSI‐H status. To explore the potential correlation between this signature and the tumor microenvironment, a series of multiplex IHC was carried out. We revealed that the stromal‐related signaling activation might contribute to an immunosuppressive environment characterized by deregulation of T cell and immunosuppressive macrophage aggregation, which thus might impair the protective factors of MSI‐H status. However, we believe that our study sheds light on the potential mechanisms by which tumor heterogeneity may influence the outcomes of the specific molecular subgroup of GC and may provide a new insight to improve the efficacy of immunotherapy.

## AUTHOR CONTRIBUTIONS

Mili Zhang, Liang Yu, Can Cao, and Jikun Li designed the research. Mili Zhang and Can Cao conducted interpretation and analysis of the data. Mili Zhang, Can Cao Performed the experiments. Mili Zhang and Liang Yu wrote, reviewed, and edited the manuscript. All authors contributed to the article and approved the submitted version.

## CONFLICT OF INTEREST

The authors declare that the research was conducted in the absence of any commercial or financial relationships that could be construed as a potential conflict of interest.

## ETHICS STATEMENT

The studies involving human samples were approved by Research Ethics Committee of the Shanghai General Hospital(IRB no.2020SQ062).

## Supporting information


Figure S1

Figure S2

Figure S3

Figure S4

Table S1

Table S2

Table S3

## Data Availability

The original contributions proposed in the study are included in the article/supplementary Materials, which can be further queried from the corresponding authors.
