# Supplementary figures and images for: Five EMT‐related genes signature predicts overall survival and immune environment in microsatellite instability‐high gastric cancer
Source: Cancer Med. 2022 Jul 4;12(2):2075–88. doi: 10.1002/cam4.4975 (PMC9883573; doi:10.1002/cam4.4975)

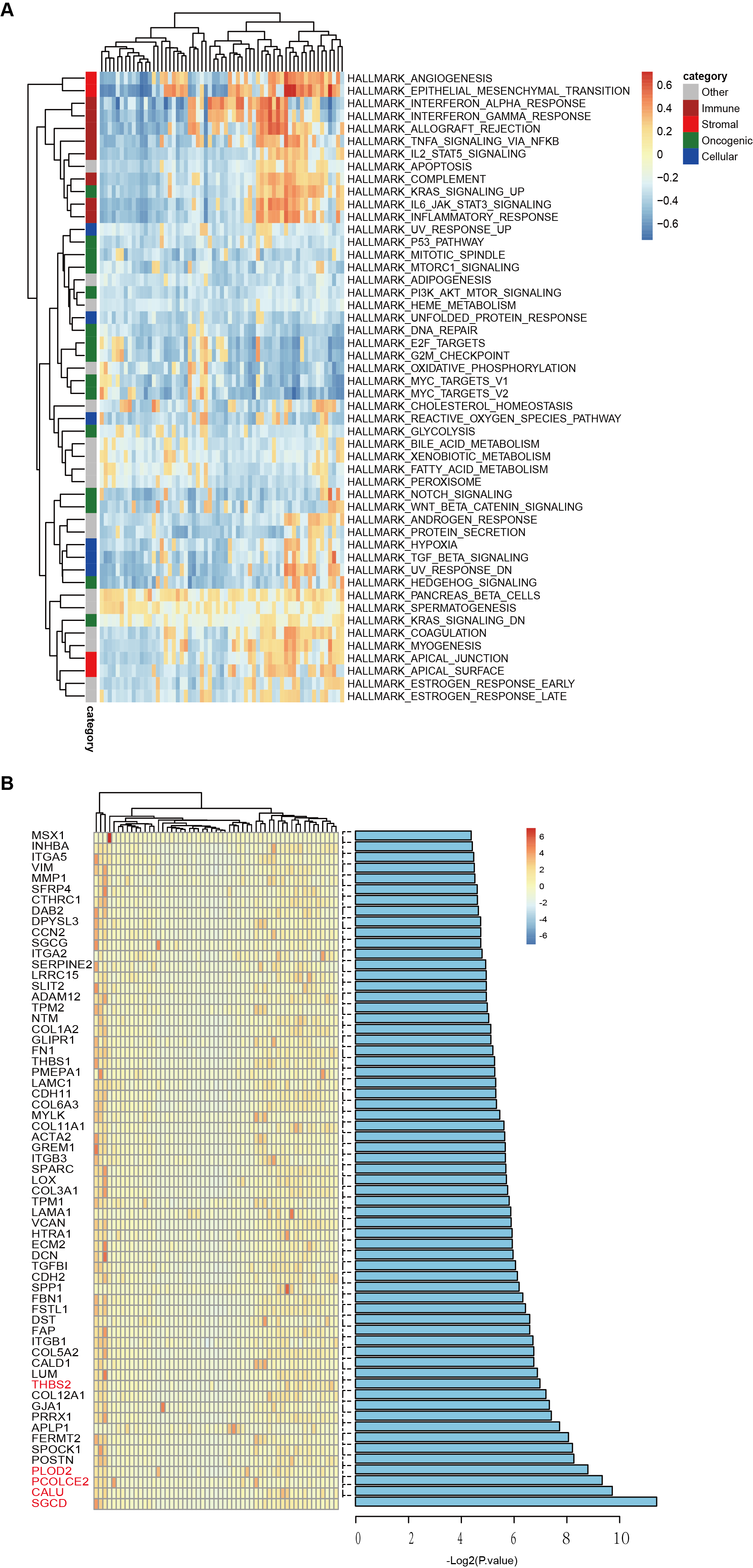

Supplement: Supplementary file 1 — Figure S1 [file CAM4-12-2075-s003.tif]

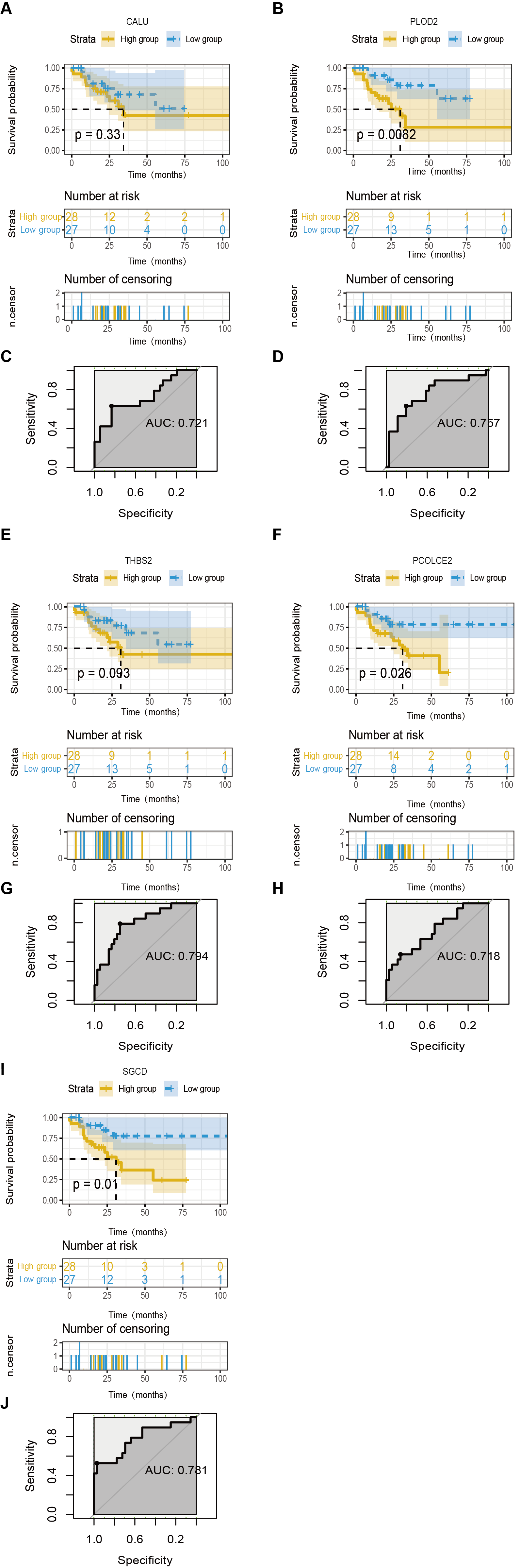

Supplement: Supplementary file 2 — Figure S2 [file CAM4-12-2075-s005.tif]

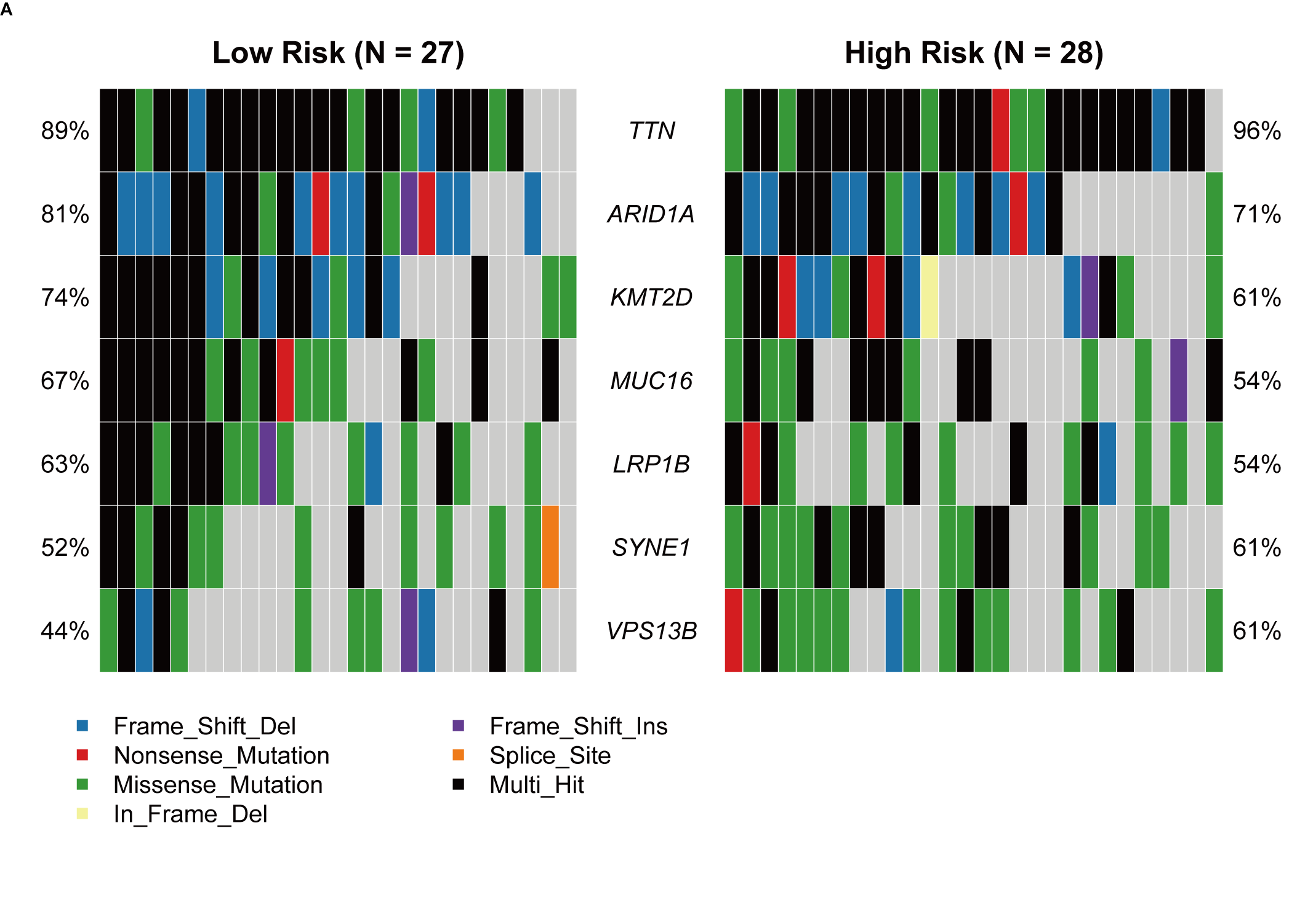

Supplement: Supplementary file 3 — Figure S3 [file CAM4-12-2075-s007.tif]

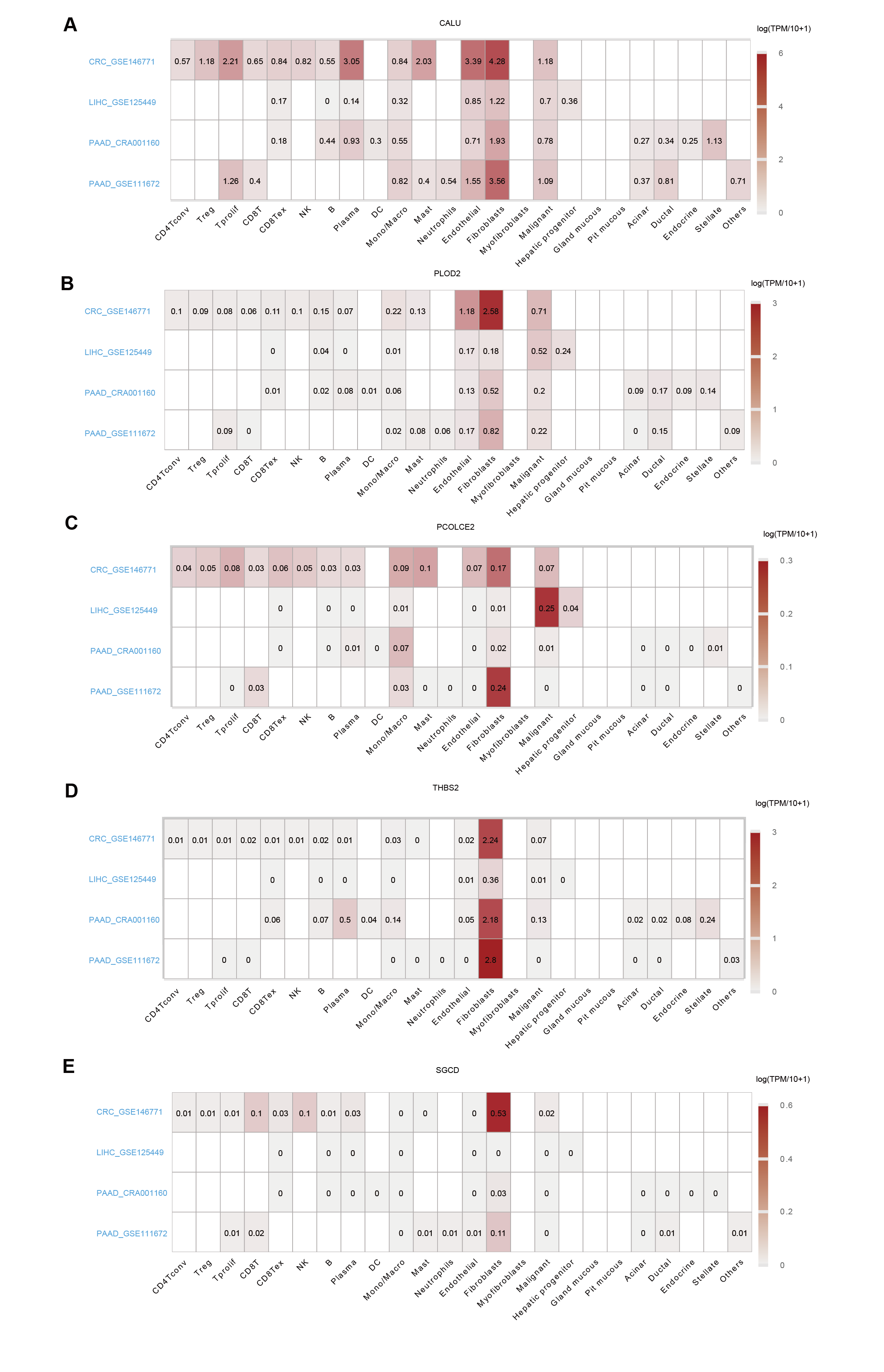

Supplement: Supplementary file 4 — Figure S4 [file CAM4-12-2075-s006.tif]
